# Uptake and Intraradical Immobilization of Cadmium by Arbuscular Mycorrhizal Fungi as Revealed by a Stable Isotope Tracer and Synchrotron Radiation μX-Ray Fluorescence Analysis

**DOI:** 10.1264/jsme2.ME18010

**Published:** 2018-09-29

**Authors:** Baodong Chen, Keiichiro Nayuki, Yukari Kuga, Xin Zhang, Songlin Wu, Ryo Ohtomo

**Affiliations:** 1 NARO Institute of Livestock and Grassland Science 768 Senbonmatsu, Nasushiobara, Tochigi, 329–2793 Japan; 2 State Key Laboratory of Urban and Regional Ecology, Research Center for Eco-Environmental Sciences, Chinese Academy of Sciences Beijing 100085 China; 3 Jilin Provincial Key Laboratory of Plant Resource Science and Green Production, Jilin Normal University Shiping 136000 China; 4 Faculty of Agriculture, Shinshu University 8304 Minami-Minowa, Kamiina, Nagano 399–4598 Japan; 5 Graduate School of Integrated Arts and Sciences, Hiroshima University 1–7–1 Kagamiyama, Higashihiroshima, Hiroshima, 739–8521 Japan

**Keywords:** arbuscular mycorrhizal fungi, cadmium, stable isotope, phytotoxicity, synchrotron radiation μX-ray fluorescence

## Abstract

Arbuscular mycorrhizal (AM) fungi can improve plant tolerance to heavy metal contamination. This detoxification ability may largely depend on how AM fungi influence the uptake and distribution of metals in host plants. Two experiments were performed in order to gain insights into the mechanisms underlying cadmium (Cd) tolerance in mycorrhizal plants. Stable isotope Cd^106^ and compartmented pots were adopted to quantify the contribution of the AM fungus, *Rhizophagus irregularis*, to the uptake of Cd by *Lotus japonicus*. Moreover, synchrotron radiation μX-ray fluorescence (SR-μXRF) was applied to localize Cd in the mycorrhizal roots at the sub-cellular level. The results obtained indicated that mycorrhizal colonization markedly enhanced Cd immobilization in plant roots. Less Cd was partitioned to plant shoots when only hyphae had access to Cd in the hyphal compartment than when roots also had direct access to the Cd pool. SR-μXRF imaging indicated that Cd absorbed by extraradical hyphae was translocated into intraradical fungal structures, in which arbuscules accumulated large amounts of Cd; however, plant cells without fungal structures and plant cell walls contained negligible amounts of Cd. The present results provide direct evidence for the intraradical immobilization of Cd absorbed by AM fungi, which may largely contribute to the enhanced tolerance of plants to Cd. Therefore, AM fungi may play a role in the phytostabilization of Cd-contaminated soil.

Cadmium (Cd) is a widespread hazardous heavy metal and its contamination has received worldwide attention (24). Various industrial and agricultural activities, including mining, metal smelting, and the application of sewage sludge to agri-cultural land, have resulted in elevated concentrations of Cd in soil (19, 25). Cd enters the food chain through crop uptake from contaminated soil, which poses a serious threat to the environment and human health. Therefore, the appropriate management of Cd in plant-soil systems and also strategies for the remediation of Cd-contaminated soil are urgently needed.

Some conventional chemical and physical methods have been applied to the remediation of Cd-contaminated soil, but are expensive or not efficient (16). Phytoremediation, a plant-based green technology, may provide a potentially feasible alternative to conventional methods (1, 31). Mcgrath *et al.* (17) reported the possibility of phytoremedation using the Zn/Cd hyperaccumulator *Thlaspi caerulescens* to remove Cd from contaminated soil. However, phytoextraction efficiency was unsatisfactory because of the slow growth rate and low biomass of hyperaccumulator plants (28). Furthermore, it did not work for large-scale Cd mining or heavily Cd-contaminated soil. Phytostabilization is an alternative strategy that reduces the mobility and bioavailability of heavy metals in soil, thereby preventing their migration into groundwater or entry into the food chain. It is simple to find plants that are suitable for phytostabilization purposes because many plants accumulate Cd in their roots despite large variations in Cd uptake capabilities (26).

An increasing number of studies have demonstrated that soil microbes, including arbuscular mycorrhizal (AM) fungi that form symbiotic associations with most higher plants, assist in the phytostabilization of Cd (7). AM fungi have been reported to establish direct links between soil and roots, and consequently improve soil nutrient exploitation by plant roots (23). AM associations are generally considered to protect plants grown in metal-contaminated soils by enhancing metal retention in roots and reducing metal partitioning to shoots (14). Cd tolerance is frequently higher in mycorrhizal plants than in non-inoculated control plants (29), and previous studies reported that mycorrhizal colonization led to an increase in the accumulation of Cd in maize roots, but a decrease in the shoots (4, 11). By using a compartmented cultivation system, Joner and Leyval (12) demonstrated that Cd^109^ added to the hyphal compartment was adsorbed by extraradical hyphae and subsequently transported to plant roots, whereas Cd transfer from the fungus to plants was restricted by fungal immobilization. Although experimental evidence has shown that mycorrhizal fungi may immobilize Cd in roots, direct evidence, such as the distribution of Cd at the cellular level in mycorrhizal roots, has not yet been obtained. One of the reasons for this is that in a commonly used method for localizing metal elements using characteristic X-rays, XRF peaks of Cd (Lα, 3.133 keV) cannot be separated from that of potassium (K) (Kα, 3.312 keV), one of the macro elements of cells. In order to eliminate the signals of biological elements and localize Cd at intracellular fungal and host structures, high energy synchrotron radiation (SR) for the detection of the Cd Kα line XRF, a cellular map analysis using the microbeam, and resin-embedded sections were combined (SR-μXRF; Nayuki *et al.* [20]).

In the present study, we performed two experiments with the aim of localizing Cd in mycorrhiza at the sub-cellular level and showing the accumulation of Cd in fungal structures. In the first experiment, the stable isotope Cd^106^ and compartmented pots were used to quantify the contribution of mycorrhiza to the uptake of Cd by *Lotus japonicus*. In the second experiment, the distributions of Cd and Zn at the cellular level in mycorrhizal roots were investigated using the SR-μXRF imaging of Cd and Zn.

## Materials and Methods

### Host plants and AM fungi

*L. japonicus* seeds were pre-germinated on moist filter paper for approximately 36 h until the appearance of radicals and were selected for uniformity before sowing. The AM fungus *Rhizophagus irregularis* (RI; DAOM197198, PremierTech, Canada; former *Glomus intraradices*) was propagated in a pot culture with maize plants grown in sandy soil for 10 weeks. The inoculum from the pot culture was a mixture of spores, mycelia, sandy soil, and root fragments, and contained approximately 200 spores 10 g^−1^ inoculum.

### Growth medium

Growth medium was loamy soil collected from an experimental plot of NILGS (NARO [National Agriculture and Food Research Organization] Institute of Livestock and Grassland Science, Nasushiobara, Tochigi, Japan, 36.92 N, 139.93 E). Soil was passed through a 2-mm sieve, autoclaved twice at 105°C for 2 h with a 2-d interval, and amended with basal nutrients without P as recommended by Pearson and Jakobsen (21). Soil had a pH of 5.2 (1:2.5 soil to water), an extractable P content of 0.13 mg kg^−1^ extracted with 0.05 mol L^−1^ HCl (hydrochloric acid)-0.025 mol L^−1^ H_2_SO_4_ (sulfuric acid), as described by Mehlich (18), and extractable Cd of 0.05 mg kg^−1^ extracted by 5 mmol L^−1^ diethylene triamine pentaacetic acid-10 mmol L^−1^ CaCl_2_-0.1 mol L^−1^ triethanolamine solution adjusted to pH 7.30, as described by Lindsay and Norvell (15).

### Compartmented cultivation system

**Fig. 1:**
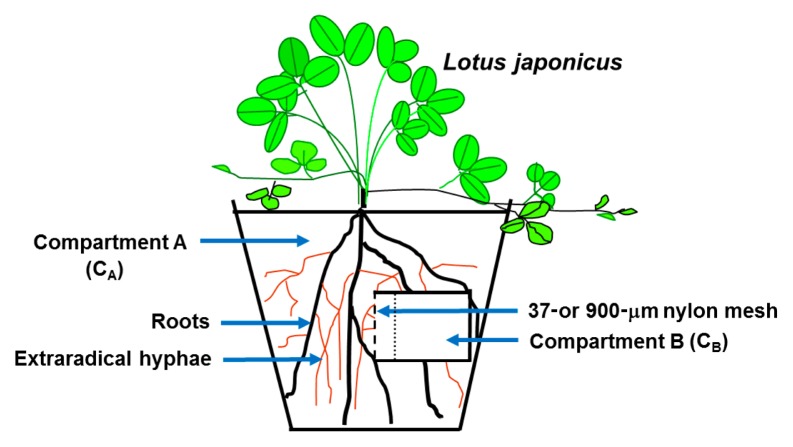
Diagram of the compartmented cultivation system in experiment I A fine (mesh size of 37 μm) or coarse (mesh size of 900 μm) nylon net was used to separate the cultivation system into two compartments: compartment A (C_A,_ plastic pot, the main compartment) for plant growth, and compartment B (C_B_, a small container buried inside the plastic pot) for hyphal (thin brown line) and root or mycorrhizal root (thick black line) development depending on the inoculation treatments and nylon net applied. There is a thin soil layer in C_B_ close to the mesh in order to prevent the diffusion of Cd over the mesh interface.

The cultivation system in experiment I was a 500-mL plastic pot constituting the main root compartment (C_A_), which contained a 30-mL plastic vial (C_B_) filled with Cd^106^-contaminated soil. The vial was sealed by a 900-μm or 37-μm mesh nylon net, which permitted the penetration of roots and hyphae or only hyphae, respectively, into contaminated soil (Fig. 1). In C_B_, there was a thin soil layer closest to the mesh (thickness of 5 mm) in order to prevent the diffusion of Cd over the mesh interface.

**Fig. 2:**
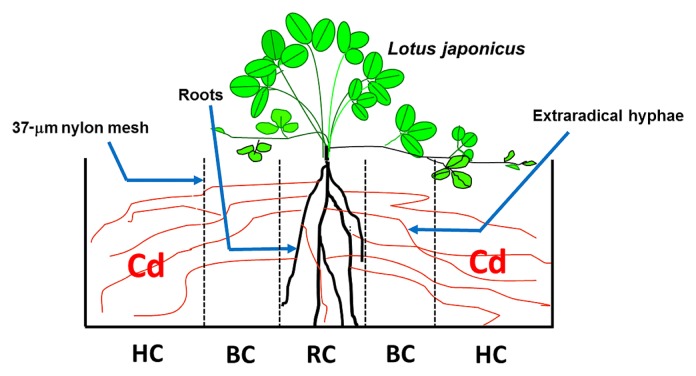
Diagram of the compartmented cultivation system in experiment II Five compartments are separated by a 37-μm nylon mesh. *Lotus japonicus* plants grown in the central root compartment (RC) were inoculated with *Rhizophagus irregularis*. The hyphal compartment (HC) received 50 mg Cd kg^−1^ and the buffer compartment (BC) was used to minimize Cd diffusion from HC to RC. In the figure, the thick black line in RC and thin brown line in all compartments indicate mycorrhizal roots and AM hyphae, respectively.

The containers in experiment II were boxes made of Plexiglas^TM^ (acrylic plate; Daicel-Evonik, Tokyo, Japan), as shown in Fig. 2. Each container was separated into five compartments by the 37-μm nylon mesh, which permitted the penetration of hyphae only. *L. japonicus* plants inoculated with RI grew in the central root compartment (RC). The hyphal compartment (HC) received Cd, and the soil buffer compartment (BC) was used to prevent the diffusion of Cd from HC to RC. Each compartment was 8 cm in height and depth, and the widths of RC, BC, and HC were 3, 2, and 4 cm, respectively (Fig. 2).

### Experimental procedure

In experiment I, nutrient-amended growth medium for each pot (C_A_) was mixed with either 25 g of the RI inoculum or an equivalent amount of the sterilized inoculum in order to obtain the inoculation treatments and non-inoculation controls. C_B_ vials received 50 g 50 mg kg^−1^ Cd^106^-contaminated soil, which was then covered by 20 g of clean soil as a buffering soil layer. The combination of the two inoculation treatments and two nylon mesh sizes resulted in 4 treatments. Each treatment had four replicates and 16 pots were set-up in a completely randomized block design. *L. japonicus* seeds were selected for uniformity before sowing, and six pre-germinated seeds were sown in each pot. Three d after emergence, seedlings were thinned to five per pot. In experiment II, 30 g of RI inoculum was mixed into each RC. A total of 50 mg Cd kg^−1^ (as Cd[NO_3_]_2_) was added to HC. Three seeds were sown, and one seedling was removed 7 d after seedling emergence.

Both experiments were conducted in a controlled environment growth chamber (KOITOTORON KG-50-HLA; Koito Manufacturing, Tokyo, Japan) with a 16-h/25°C d and 8-h/18°C night under artificial light. During the experimental period, deionized water was added to maintain a soil moisture content of 15% on a dry soil basis (approximately 55% water holding capacity) by regular weighing.

### Harvest and analysis

The first experiment was harvested 8 weeks after seedling emergence. Shoots carefully cut off from the roots in C_A_ were washed with tap water. All root samples were carefully washed with deionized water to remove adhering soil particles. Sub-samples of fresh roots were collected for the assessment of AM colonization. Fresh roots were cleared in 10% KOH and stained with Trypan blue by a modified procedure of Phillips and Hayman (22), omitting phenol from solutions and HCl from the rinse. Percentage root colonization and root length were assessed using the grid line intersect method (8). The dry weights of shoots and roots were measured after oven drying at 70°C for 48 h. Oven-dried sub-samples were milled and 100 mg of samples were acid digested with nitric acid and dissolved in 10 mL of 0.5 mol L^−1^ nitric acid. In addition to the samples, four procedure blanks were included.

Dissolved samples were analyzed for cadmium (Cd^106^) using inductively coupled plasma-mass spectrometry (ICP-MS, 7500C; Agilent Technologies, Tokyo, Japan). Phosphorus (P) concentrations in the digested samples were assessed as described by Chen *et al.* (5) with modifications. Fifty microliters of solution was reacted in a final volume of 200 μL of molybdenum-blue assay solution (0.252% [NH_4_]_6_Mo_7_O_24_·4H_2_O [hexaammonium heptamolybdate tetrahydrate], 200 mmol L^−1^ H_2_SO_4_, 1% ascorbate) at 45°C for 20 min, and absorbance at 820 nm was measured using the microplate reader Multiskan Ascent (Thermo Labsystems, Helsinki, Finland).

In the SR-μXRF analysis (experiment II), compartmented pots were dismantled at harvest and the shoots and roots of *L. japonicus* plants were sampled separately. Root samples were carefully washed with deionized water to remove adhering soil particles and were cut in half under a binocular microscope to select the root pieces colonized. The remainder of the root and shoot samples were analyzed for Cd concentrations by ICP-MS. The selected root pieces were then processed for high pressure freezing, freeze-substituted by dried acetone, embedded in Spurr’s resin, and polymerized at 60°C overnight (20). Resin-embedded materials were semi-thin sectioned using an ultramicrotome (Leica EM UC7; Vienna, Austria). Sections were stained with toluidine blue O (TBO) for structural observations under a light microscope or stained with 4′,6-diamidino-2-phenylindole (DAPI) for the observation of polyphosphate by yellow fluorescent emission under UV excitation (LP420 filter; Zeiss Axio ImagerA1). A serial thick section (thickness of 10 μm) was prepared for the SR-μXRF analysis according to Nayuki *et al.* (20). Briefly, the section was fixed on a drop of water placed on a polypropylene membrane (PP, 6-μm-thick roll sheet; Rigaku Corporation, Tokyo, Japan), and the section on the membrane was attached at the center of a hole in the acrylic plate (40×40 mm, thickness of 1 mm, hole with a diameter of 5 mm.). The acrylic holder was attached to the magnetic holder and Cd and Zn mapping was analyzed at SR-μXRF using SPring-8 BL37XU (Hyogo, Japan). The distribution of Zn was measured to obtain structural information on the analyzed area because Zn was previously shown to localize in host and fungal cell walls (20). Detailed machinery configurations and the settings of the beam line are described elsewhere (10). Excitation X-ray energy was 30 keV, the beam size was 1.3×0.86 μm, and energy ranges (keV) for detecting the Cd Kα line and Zn Kα line were 22.60–23.53 and 8.38–8.87, respectively. μXRF spectra were measured at arbuscules (live, expanded arbuscules with fine branches, or dead, a collapsed mass of fine branches), intercellular hyphae, vesicles (cell wall and inside), and root cells (cell wall and inside) at 300 s.

### Statistical analysis

Data from experiment I were subjected to a two-way ANOVA in order to compare the mycorrhizal status and mesh size using SPSS 16.0 (IBM Corporation, Armonk, NY, USA). The significance of differences between means was assessed using Tukey’s HSD test at a probability level of 0.05.

## Results

### Mycorrhizal colonization, plant growth, and phosphorus nutrition

**Fig. 3:**
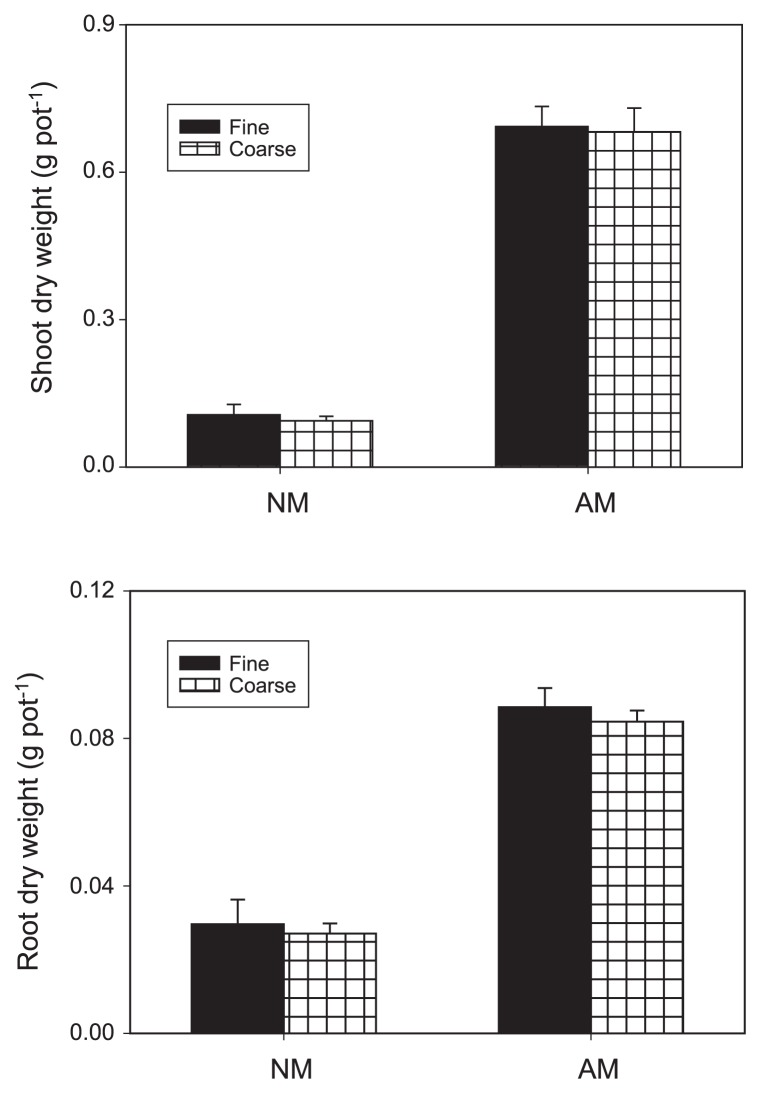
Shoot and root dry weights of *Lotus japonicus* under different treatments NM and AM represent the non-inoculated treatment and inoculation with the mycorrhizal fungus *Rhizophagus irregularis*. Error bars indicate standard deviations estimated from 4 replicated experiments. In the analysis of variance, the inoculation was significant (*P*<0.001) for both the shoots and roots. The mesh size of the nylon net did not have a significant effect on the shoots or roots (*P*>0.05), and the interaction of the inoculation with the mesh size also had no significant effect (*P*>0.05).

**Fig. 4:**
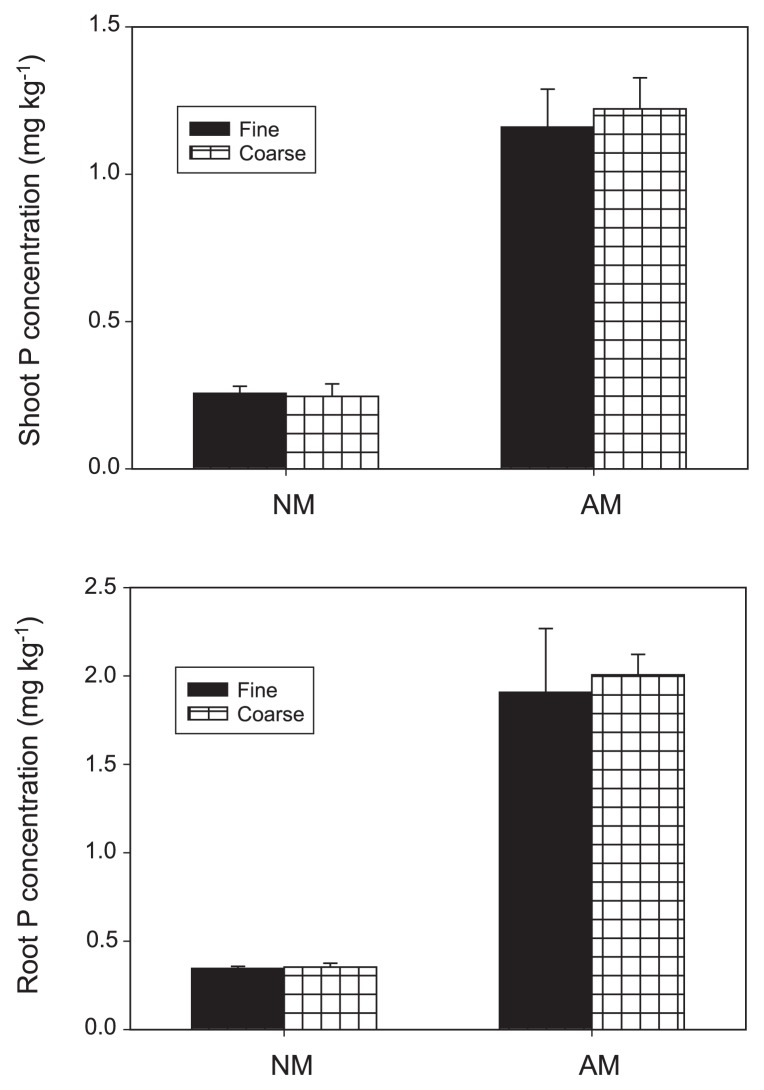
Shoot and root phosphate concentrations of *Lotus japonicus* under different treatments NM and AM represent the non-inoculated treatment and inoculation with the mycorrhizal fungus *Rhizophagus irregularis*. Error bars indicate standard deviations estimated from 4 replicated experiments. In an analysis of variance, the inoculation was significant (*P*<0.001) for both the shoots and roots. The mesh size of the nylon net did not have a significant effect on the shoots or roots (*P*>0.05), and the interaction of the inoculation with the mesh size also showed no significant effect (*P*>0.05).

In experiment I, the roots of inoculated plants were extensively colonized by RI, while non-inoculated controls remained uncolonized (Table 1). The mesh size had no significant effect on mycorrhizal colonization. The dry weights of the shoots and roots were significantly increased by the inoculation with RI (*P*<0.001), but were unaffected by compartmentation (Fig. 3). Mesh size had no significant effect on the root to shoot ratio of plant dry weights, whereas mycorrhizal colonization decreased the root to shoot ratio of plant dry weights (*P*<0.001, Table 1). Mycorrhizal colonization increased plant P concentrations in the shoots and roots (*P*<0.001, Fig. 4), while shoot and root P concentrations were not affected by the mesh size.

### Cd uptake and partitioning

**Fig. 5:**
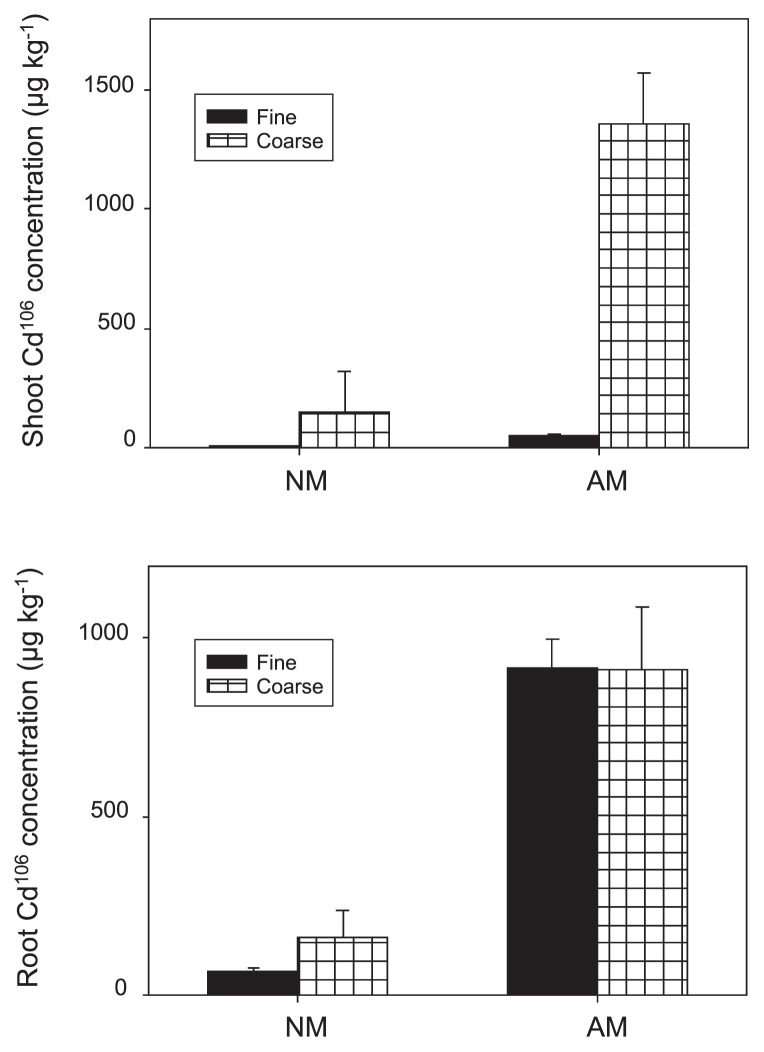
Shoot and root Cd^106^ concentrations of *Lotus japonicus* under different treatments NM and AM represent the non-inoculated treatment and inoculation with the mycorrhizal fungus *Rhizophagus irregularis*. Error bars indicate standard deviations estimated from 4 replicated experiments. In an analysis of variance, the inoculation was significant (*P*<0.001) for both the shoots and roots. The shoot Cd^106^ concentration was also significantly influenced by the mesh size of the nylon net (*P*<0.001) and the interaction of the inoculation with the mesh size (*P*<0.001). Neither the mesh size nor the interaction of the inoculation with the mesh size had significant effects on the root Cd^106^ concentration (*P*>0.05).

Shoot Cd^106^ concentrations in inoculated plants (μg Cd kg^−1^ shoot dry weight) was significantly increased by root penetration into Cd-contaminated C_B_ soil (*P*<0.001, Fig. 5), while shoot Cd^106^ concentrations in inoculated plants with fine mesh sealing were not significantly different from those in non-inoculated controls. As a result, a significant interaction effect of the inoculation and mesh size (*P*<0.001) was observed for shoot Cd^106^ concentrations. Mycorrhizal colonization significantly increased root Cd^106^ concentrations (*P*<0.001, Fig. 5). Root Cd^106^ concentrations were not significantly different irrespective of a fine or coarse mesh separating C_B_ from C_A_.

**Fig. 6:**
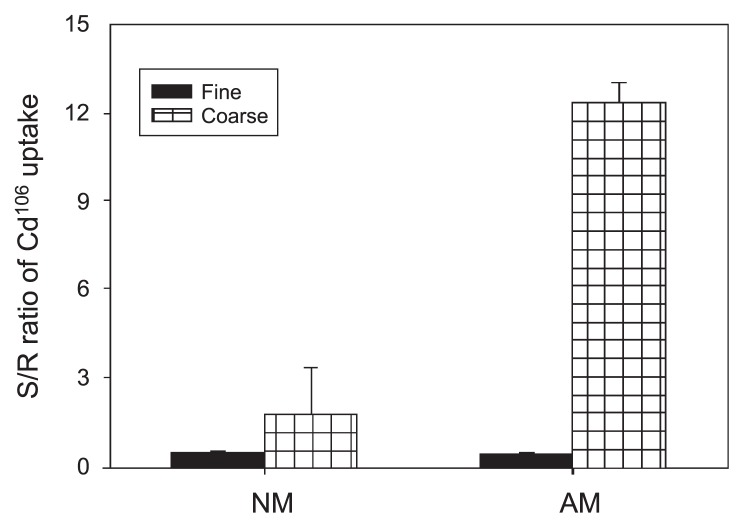
Shoot to root ratio of Cd^106^ uptake by *Lotus japonicus* as influenced by different treatments NM and AM represent the non-inoculated treatment and inoculation with the mycorrhizal fungus *Rhizophagus irregularis*. Error bars indicate standard deviations estimated from 4 replicated experiments. In an analysis of variance, the inoculation was highly significant (*P*<0.001) for the shoot to root (S/R) ratio of Cd^106^ uptake. The S/R ratio of Cd^106^ uptake was also significantly influenced by the mesh size of the nylon net (*P*<0.001). No significant interaction was detected between the inoculation and mesh size (*P*>0.05).

The amount of Cd^106^ (*i.e.*, concentration [Fig. 5]×dry weight [Fig. 3]) in the shoots of inoculated plants in the fine mesh treatment was half of that in the roots (Fig. 6), indicating only 1/3 of total Cd^106^ uptake partitioned from the roots to shoots. This value was significantly lower than that with root access to C_B_ (*P*<0.001), but was not significantly different from the non-inoculated treatments.

### Subcellular distribution of Cd in mycorrhizal roots (Experiment II)

**Fig. 7:**
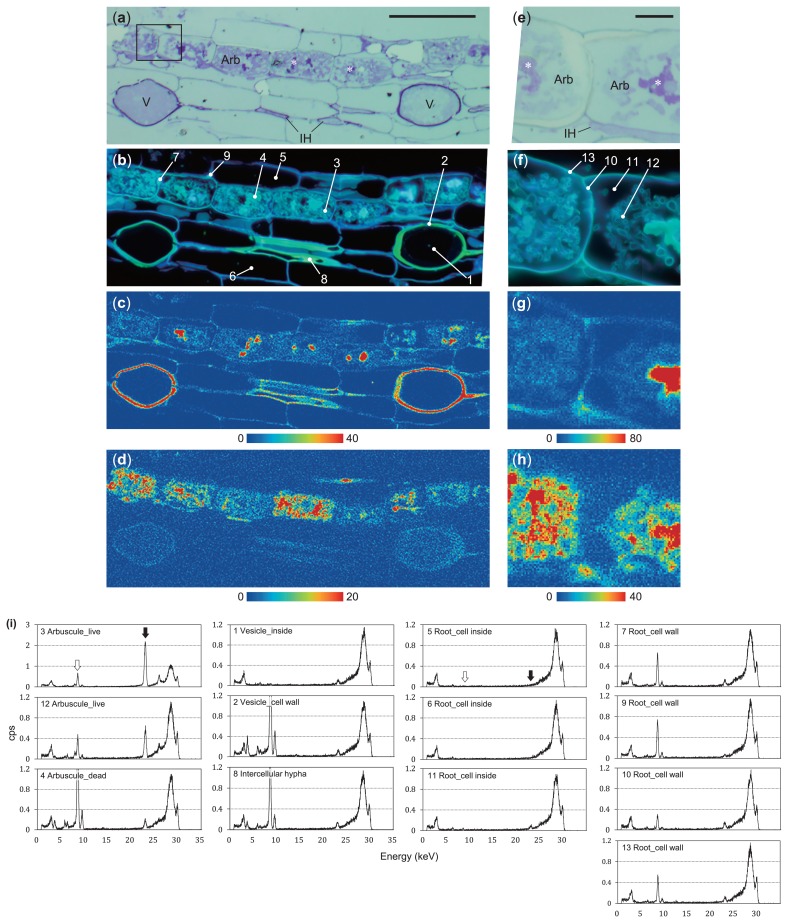
Synchrotron radiation μX-ray fluorescence (SR-μXRF) images of resin sections of arbuscular mycorrhiza to which Cd was added to hyphal compartments (a)–(d): A longitudinal section of a colonized root. (e)–(h): A part of (a)–(d) shown in a square in A. TBO staining (a & e) and DAPI staining (b & f) of serial sections of SR-μXRF. Zn (c & g) and Cd (d & h) distributions were taken from the same section. (i): SR-μXRF spectra shown as counts s^−1^ taken at the points shown in (b) and (f) with numbers (measurement time, 300 s). Zn accumulated in the cell walls of plant and fungal cells, particularly in dead arbuscules (indicated with asterisks) and the cell walls of vesicles, thereby proving cell structural information. Cd was distributed in fungal structures only, particularly in live arbuscules and intraradical hyphae. Arb, arbuscule; V, vesicle; IH, intercellular hypha. Bar: (a)–(d), 100 μm; (e)–(h), 10 μm. Scanning condition for maps: c & d, step of 1 μm, exposure time of 0.5 sec; (g) & (h), step of 0.5 μm, exposure time of 1 s. The corresponding positions of Zn Kα (8.632 keV) and Cd Kα (23.11 keV) lines are shown in the white and black arrows in (i), respectively.

Shoot and root Cd^106^ concentrations in the plants used in experiment II were 1.5 and 51.6 mg kg^−1^, respectively. The distributions of Cd and Zn in longitudinal sections of mycorrhizal roots are shown in Fig. 7. Zn originating from soil, namely, absorbed by both the roots and hyphae, accumulated in the cell walls of root and fungal cells. The structures that largely accumulated Zn were dead arbuscules, the cell walls of vesicles, intercellular hyphae, and root cell walls. Cd, absorbed by extraradical hyphae only, mainly localized in fungal structures. Arbuscules and intercellular hyphae accumulated large amounts of Cd, followed by the vesicles (Fig. 7). In contrast, plant cell structures did not show the accumulation of Cd, which appeared as a weak (Point 10 in Fig. 7) or trace signal (Point 9 in Fig. 7) in the spectra of host cell walls, even in colonized cells.

## Discussion

In experiment I, the compartmented cultivation system was used in an attempt to separate and compare Cd uptake by extraradical AM hyphae and plant roots from stable isotope-labeled soil. Since C_B_ only occupied 6% of the volume of C_A_, this investigation was under nearly natural conditions without marked disturbances by the compartmentation design. The thin soil layer in C_B_ closest to the mesh prevented the diffusion of Cd over the mesh interface. Furthermore, the isotope Cd^106^ in C_B_ allowed for the tracing of Cd uptake by hyphae and roots without interference from background Cd in natural soil. Therefore, this experiment ensured the reliability of data to compare the uptake and translocation of Cd by roots and AM hyphae.

By using this cultivation system, we found that the immobilization of Cd in roots was markedly enhanced by mycorrhizal colonization, which was shown by a higher root Cd concentration in inoculated plants (Fig. 5), as reported previously by Joner and Leyval (12). Furthermore, more Cd was partitioned to plant shoots when a coarse nylon net (900 μm) was used rather than a fine mesh (37 μm) allowing only hyphal access to C_B_. This was indicated by the markedly higher shoot to root ratio of total Cd uptake in the inoculated plants when the coarse mesh was used (Fig. 6), implying that Cd taken up through the root pathway translocates more easily to plant shoots than that through the fungal pathway. Mycorrhizal-mediated Cd uptake was most likely immobilized in fungal structures or the mycelium (12). Joner *et al.* (13) previously reported that isolated AM extraradical mycelia had a high sorption capacity of Cd. Similarly, the high affinity of the fungal mycelium for other heavy metals, such as Zn, was also confirmed (2).

Previous studies demonstrated that mycorrhizal associations may reduce the accumulation of certain heavy metals in the shoots of host plants, presumably by enhancing metal retention within roots (3, 6, 7, 30, 32). By using a chemical extraction method, Wang *et al.* (27) investigated the impact of AM fungi on the subcellular distribution and chemical forms of Cd in alfalfa and found that mycorrhizal inoculations increased Cd in the cell walls of host plants, and the proportion of the inactive forms of Cd in roots was higher in mycorrhizal plants. However, direct evidence for the immobilization of Cd in mycorrhizal roots by AM fungi is still lacking. González-Guerrero *et al.* (9) used an electron dispersive X-ray synchrotron (EDS) to localize Zn, Cu, and Cd in the extraradical mycelia and spores of *G. intraradices* and found that Zn, Cu, and Cd mainly accumulated in fungal cell walls and vacuoles. Nevertheless, EDS is not suitable for the detection of Cd in biological materials because the energy peak of the characteristic X-ray fluorescence (XRF) of Cd (Lβ1, 3.316 keV) overlapped with that of K (Kα, 3.312 keV). In contrast, high-energy SR detects Cd by K line XRF (23.11 keV), which eliminates the possibility of signal contamination from co-existing lighter elements. Therefore, SR-μXRF of SPring-8 (BL37XU) is regarded as an ideal tool to study the distribution of elements in live plants (20).

In experiment II, high pressure freezing techniques were used to localize Cd in mycorrhizal tissues in order to minimize structural and elemental re-location and replacement, and μXRF was then applied to investigate the distribution of Cd in mycorrhiza. SR-μXRF imaging revealed that the arbuscules and intercellular hyphae accumulated large amounts of Cd (Fig. 7), followed by the vesicles (Fig. 7), while plant cells did not. This distribution pattern suggested that after the extraradical hyphae uptake and translocation of Cd to intraradical hyphae, this toxic metal was mainly retained in the fungal structure, particularly in the arbuscules, and did not appear to be delivered to plant cells. These results explain those from experiment I showing that Cd uptake through the fungal pathway was not easily translocated to shoots. Furthermore, the present results provided direct evidence for the intraradical immobilization of Cd by AM fungi, which largely contributed to the protective effects of the mycorrhizal association on host plants against Cd contamination.

In summary, our investigation strongly supports the direct uptake and transport of Cd by AM fungi, and revealed in detail the spatial distribution of Cd in mycorrhizal roots following fungal uptake. The present results contribute to a deeper understanding of the protective effects of mycorrhizal fungi on host plants exposed to metal contamination. Furthermore, the present study highlighted the potential role of AM fungi in the future bioremediation of Cd-contaminated environments.

## Figures and Tables

**Table 1:** Mycorrhizal colonization rate and root to shoot ratio of *Lotus japonicus* plants in association with *Rhizophagus irregularis*.

Inoculation treatment	Type of nylon mesh	Root colonization (%)	Root to shoot ratio
Non-inoculated	Fine	0	0.28
	Coarse	0	0.29
Inoculated	Fine	45	0.13
	Coarse	47	0.12
Significance^a^ of			
Inoculation (I)		***	***
Mesh type (M)		NS	NS
I×M		NS	NS

a By a two-way analysis of variance;

*** *P*<0.001;

**P<0.01;

*P<0.05;

NS—not significant.

## References

[1] Ali H., Khan E., Sajad M.A. (2013). Phytoremediation of heavy metals—concepts and applications. Chemosphere.

[2] Chen B., Christie P., Li X. (2001). A modified glass bead compartment cultivation system for studies on nutrient and trace metal uptake by arbuscular mycorrhiza. Chemosphere.

[3] Chen B.D., Li X.L., Tao H.Q., Christie P., Wong M.H. (2003). The role of arbuscular mycorrhiza in zinc uptake by red clover growing in a calcareous soil spiked with various quantities of zinc. Chemosphere.

[4] Chen B.D., Liu Y., Shen H., Li X.L., Christie P. (2004). Uptake of cadmium from an experimentally contaminated calcareous soil by arbuscular mycorrhizal maize (Zea mays L.). Mycorrhiza.

[5] Chen P.S., Toribara T.Y., Warner H. (1956). Microdetermination of phosphorus. Anal Chem (Washington, DC, U S).

[6] Christie P., Li X., Chen B. (2004). Arbuscular mycorrhiza can depress translocation of zinc to shoots of host plants in soils moderately polluted with zinc. Plant Soil.

[7] Gaur A., Adholeya A. (2004). Prospects of arbuscular mycorrhizal fungi in phytoremediation of heavy metal contaminated soils. Curr Sci.

[8] Giovanetti M., Mosse B. (1980). An evaluation of techniques for measuring vesicular arbuscular mycorrhizal infection in roots. New Phytol.

[9] González-Guerrero M., Melville L.H., Ferrol N., Lott J.N.A., Azcón-Aguilar C.n., Peterson R.L. (2008). Ultrastructural localization of heavy metals in the extraradical mycelium and spores of the arbuscular mycorrhizal fungus *Glomus intrararadices*. Can J Microbiol.

[10] Hokura A., Onuma R., Kitajima N., Terada Y., Saito H., Abe T., Yoshida S., Nakai I. (2006). 2-D X-ray fluorescence imaging of cadmium hyperaccumulating plants by using high-energy synchrotron radiation X-ray microbeam. Chem Lett.

[11] Huang H., Zhang S., Chen B.-D., Wu N., Shan X.-Q., Christy P. (2006). Uptake of atrazine and cadmium from soil by maize (*Zea mays* L.) in association with the arbuscular mycorrhizal fungus *Glomus etunicatum*. J Agric Food Chem.

[12] Joner E.J., Leyval C. (1997). Uptake of ^109^Cd by roots and hyphae of a *Glomus mosseae*/*Trifolium subterraneum* mycorrhiza from soil amended with high and low concentrations of cadmium. New Phytol.

[13] Joner E.J., Briones R., Leyval C. (2000). Metal-binding capacity of arbuscular mycorrhizal mycelium. Plant Soil.

[14] Leyval C., Joner E.J., Gobran G.R., Wenzel W.W., Lombi E. (2001). Bioavailability of heavy metals in the mycorrhizosphere. Trace Elements in the Rhizosphere.

[15] Lindsay W.L., Norvell W.A. (1978). Development of DTPA soil test for Zinc, Iron, Manganese, and Copper. Soil Sci Soc Am J.

[16] Loganathan P., Vigneswaran S., Kandasamy J., Naidu R. (2012). Cadmium sorption and desorption in soils: A review. Crit Rev Environ Sci Technol.

[17] McGrath S.P., Lombi E., Gray C.W., Caille N., Dunham S.J., Zhao F.J. (2006). Field evaluation of Cd and Zn phytoextraction potential by the hyperaccumulators Thlaspi caerulescens and Arabidopsis halleri. Environ Pollut.

[18] Mehlich A. (1953). Rapid determination of cation and anion exchange properties and pH of soils. J Assoc Off Anal Chem.

[19] Nawrot T., Plusquin M., Hogervorst J., Roels H.A., Celis H., Thijs L., Vangronsveld J., Van Hecke E., Staessen J.A. (2006). Environmental exposure to cadmium and risk of cancer: a prospective population-based study. Lancet Oncol.

[20] Nayuki K., Chen B., Ohtomo R., Kuga Y. (2014). Cellular imaging of cadmium in resin sections of arbuscular mycorrhizas using synchrotron micro X-ray fluorescence. Microbes Environ.

[21] Pearson J.N., Jakobsen I. (1993). The relative contribution of hyphae and roots to phosphorus uptake by arbuscular mycorrhizal plants, measured by dual labelling with ^32^P and ^33^P. New Phytol.

[22] Phillips J.M., Hayman D.S. (1970). Improved procedures for clearing roots and staining parasitic and vesicular-arbuscular mycorrhizal fungi for rapid assessment of infection. Trans Br Mycol Soc.

[23] Smith S.E., Read D. (2008). Mycorrhizal Symbiosis.

[24] Toppi L.S.d, Gabbrielli R. (1999). Response to cadmium in higher plants. Environ Exp Bot.

[25] Vassilev A., Vangronsveld J., Yordanov I. (2002). Cadmium phytoextraction: Present state, biological backgrounds and research needs. Bulg J Plant Phys.

[26] Wahid A., Arshad M., Farooq M., Lichtfouse E. (2010). Cadmium phytotoxicity: Responses, mechanisms and mitigation strategies: A review. Organic Farming, Pest Control and Remediation of Soil Pollutants.

[27] Wang Y., Huang J., Gao Y. (2012). Arbuscular mycorrhizal colonization alters subcellular distribution and chemical forms of cadmium in Medicago sativa L. and resists cadmium toxicity. PLoS One.

[28] Wu G., Kang H., Zhang X., Shao H., Chu L., Ruan C. (2010). A critical review on the bio-removal of hazardous heavy metals from contaminated soils: issues, progress eco-environmental concerns and opportunities. J Hazard Mater.

[29] Zhang X., Chen B., Ohtomo R. (2015). Mycorrhizal effects on growth, P uptake and Cd tolerance of the host plant vary among different AM fungal species. Soil Sci Plant Nutr.

[30] Zhang X.-H., Lin A.-J., Gao Y.-L., Reid R.J., Wong M.-H., Zhu Y.-G. (2009). Arbuscular mycorrhizal colonisation increases copper binding capacity of root cell walls of Oryza sativa L. and reduces copper uptake. Soil Biol Biochem.

[31] Zhao F.J., McGrath S.P. (2009). Biofortification and phytoremediation. Curr Opin Plant Biol.

[32] Zhu Y.G., Christie P., Laidlaw A.S. (2001). Uptake of Zn by arbuscular mycorrhizal white clover from Zn-contaminated soil. Chemosphere.

